# Identification and molecular characterization of novel duck reoviruses in Henan Province, China

**DOI:** 10.3389/fvets.2023.1137967

**Published:** 2023-03-30

**Authors:** Zhifeng Peng, Han Zhang, Xiaozhan Zhang, Haiyan Wang, Zihan Liu, Hongxing Qiao, Yujin Lv, Chuanzhou Bian

**Affiliations:** ^1^College of Veterinary Medicine, Henan University of Animal Husbandry and Economy, Zhengzhou, China; ^2^College of Animal Science and Veterinary Medicine, Henan Institute of Science and Technology, Xinxiang, China

**Keywords:** duck spleen necrosis disease, NDRV, genome sequence, epidemiology, evolutionary

## Abstract

Novel Duck reovirus (NDRV) is an ongoing non-enveloped virus with ten double-stranded RNA genome segments that belong to the genus *Orthoreovirus*, in the family *Reoviridae*. NDRV-associated spleen swelling, and necrosis disease have caused considerable economic losses to the waterfowl industry worldwide. Since 2017, a significant number of NDRV outbreaks have emerged in China. Herein, we described two cases of duck spleen necrosis disease among ducklings on duck farms in Henan province, central China. Other potential causative agent, including Muscovy duck reovirus (MDRV), Duck hepatitis A virus type 1 (DHAV-1), Duck hepatitis A virus type 3 (DHAV-3), Newcastle disease virus (NDV), and Duck tembusu virus (DTMUV), were excluded by reverse transcription-polymerase chain reaction (RT-PCR), and two NDRV strains, HeNXX-1/2021 and HNJZ-2/2021, were isolated. Sequencing and phylogenetic analysis of the σC genes revealed that both newly identified NDRV isolates were closely related to DRV/SDHZ17/Shandong/2017. The results further showed that Chinese NDRVs had formed two distinct clades, with late 2017 as the turning point, suggesting that Chinese NDRVs have been evolving in different directions. This study identified and genetic characteristics of two NDRV strains in Henan province, China, indicating NDRVs have evolved in different directions in China. This study provides an insight into the ongoing emerged duck spleen necrosis disease and enriches our understanding of the genetic diversity and evolution of NDRVs.

## 1. Introduction

Avian reovirus (ARV) has caused immense economic problems in the chicken, duck, goose, and turkey industries worldwide (1, 2). Waterfowl reoviruses (WRVs) have been associated with various disease conditions in ducks and geese of different species, including Muscovy duck white spot disease caused by classical Muscovy duck reovirus (MDRV) (3–7), duck hemorrhagic necrotizing hepatitis, and spleen necrosis disease in ducks caused by the novel Muscovy duck reovirus (N-MDRV) (8–10) and NDRV (8, 10–15), spleen and liver inflammation in geese caused by goose reovirus (GRV) (1, 3, 16), hemorrhagic necrotic hepatitis caused by the new type of goose reovirus (N-GRV) (17). It has been reported that WRVs can be classified into two genotypes (16). Genotype 1 comprises MDRV and GRV. While genotype 2 comprises NDRV, N-MDRV, and N-GRV (16).

The ARV genome includes ten segments that can be separated into three classes based on their sizes: large (L1, L2, L3), middle (M1, M2, M3), and small (S1, S2, S3, S4). The S1 segment is the only tricistronic gene that encodes P10, P18, and σC proteins (13, 18). The outer capsid proteins σC, σB, and μB of ARV are considerably variable, whereas the inner capsid proteins are relatively conservative (18). Additionally, σC proteins play an essential role in viral fusion, invasion, neutralizing antibody induction, and pathogenicity (18–21). Meanwhile, σC is regarded as the most variable protein of all the ARV proteins (18). σC gene was usually used for epidemiological studies and viral classification of ARVs (15, 22).

In recent years, many NDRV outbreaks emerged in China (10, 23–25). However, few studies have performed the evolutionary status analysis on NDRV in central China. In this study, we isolated two NDRV strains from different duck farms in Henan province, central China. To better understand the molecular characteristics of the NDRVs circulating in duck populations, the σC genes of both isolates were cloned, sequenced, and their phylogeny and mutations were analyzed. This study systematically describes the genetic and evolutionary characteristics of the ongoing NDRV strains and highlights that continuous surveillance is needed to develop proper vaccines and reasonable control programs.

## 2. Materials and methods

### 2.1. Sampling cases presentation

#### 2.1.1. Case 1

In March 2021, acute outbreaks of spleen necrosis disease occurred on many commercial duck farms in Xinxiang city of Henan province, central China. A duck farm with approximately 30% of 10,000 10-day-old ducklings showed sudden onset and severe symptoms, such as listlessness, white diarrhea, anorexia, and lameness. The outbreak started on 3 March 2021, and antibiotic-traditional veterinary drugs combination therapy did not work. Seven dead ducklings were randomly selected and sent to the laboratory for diagnosis.

#### 2.1.2. Case 2

An epidemic characterized by the sudden death of ducklings emerged on another commercial duck farm in Jiaozuo city of Henan province. The duck flock had approximately 11,000 ducklings of 11-day-old. From 12 August 2021 to 22 August 2021, approximately 350 ducklings per day died acutely. The great majority of the diseased ducklings showed listlessness, white diarrhea, and anorexia. The mortality was about 30%. The antibiotic therapy did not work. Eight dead ducklings were selected randomly to send to the laboratory for diagnosis.

### 2.2. Molecular diagnosis

To identify the causative agent of the disease, the potential viral and bacterial pathogens were examined. The bacteriological culture was performed as described previously (26). The samples from the same duckling were combined, and processed by extracting RNA using the EasyPure Viral RNA Kit (Takara, Shanghai). Subsequently, the RNA samples were used to detect MDRV, DHAV-1, DHAV-3, NDV, DTMUV, and NDRV using RT-PCR protocols as described previously (Supplementary Table S1) (27). The PCR products were collected by electrophoresis in 1.0% agarose gel with DL2000 DNA Ladder (Takara, Shanghai).

### 2.3. Virus isolation

The positive samples of liver and spleen from dead ducklings from the same flock were homogenized in phosphate-buffered saline (PBS, pH 7.2), freeze-thawed three times, and centrifuged at 8,000 × g for 10 min. The supernatants were filtered through a 0.22 μm filter to remove bacteria and other larger particles. Subsequently, 0.2 mL of each of the two supernatants was separately used to propagate in the allantoic cavity of 10-day-old healthy duck embryos in a 37°C incubator. If the embryo died at 3–4 days post-inoculation, the allantoic fluid was harvested for another round of inoculation. After three passages in healthy embryonated duck eggs, the allantoic fluids and duck embryos were harvested sterilely and stored at −80°C. The viral RNA extracted from the allantoic fluids were used to detect potential causative agent.

### 2.4. Gene amplification and sequencing

To analyze the genotype and genetic characteristics of the both newly isolated NDRV strains in this study, the complete σC genes of the NDRV were amplified by using primers as follows: NDRV-S1 forward: 5′-GCTTTTTTCTTCTCTGCCCAT-3′ and NDRV-S1 reverse: 5′- GATGAATAGCTCTTCTCATCGC-3′, which were designed based on the S1 gene of NDRV downloaded from NCBI (https://www.ncbi.nlm.nih.gov/). The RT-PCR products were purified and cloned into a pMD18-T vector (Takara, Shanghai) for sequencing with universal M13 forward and reverse primers by Sangon Biotech in Shanghai.

### 2.5. Phylogenetic analysis and sequence comparison

The σC genes of both newly identified NDRV isolates were subjected to the online BLAST program (https://blast.ncbi.nlm.nih.gov/Blast.cgi). And sequences sharing more than 95% nucleotide identity were downloaded for further genetic analysis with Clustal Omega. Furthermore, the phylogenetic tree was conducted by MEGA 6.0 software with the neighbor-joining method using 1,000 bootstrap replicates.

## 3. Results

### 3.1. Clinical signs and post-mortem examinations

**Figure 1:**
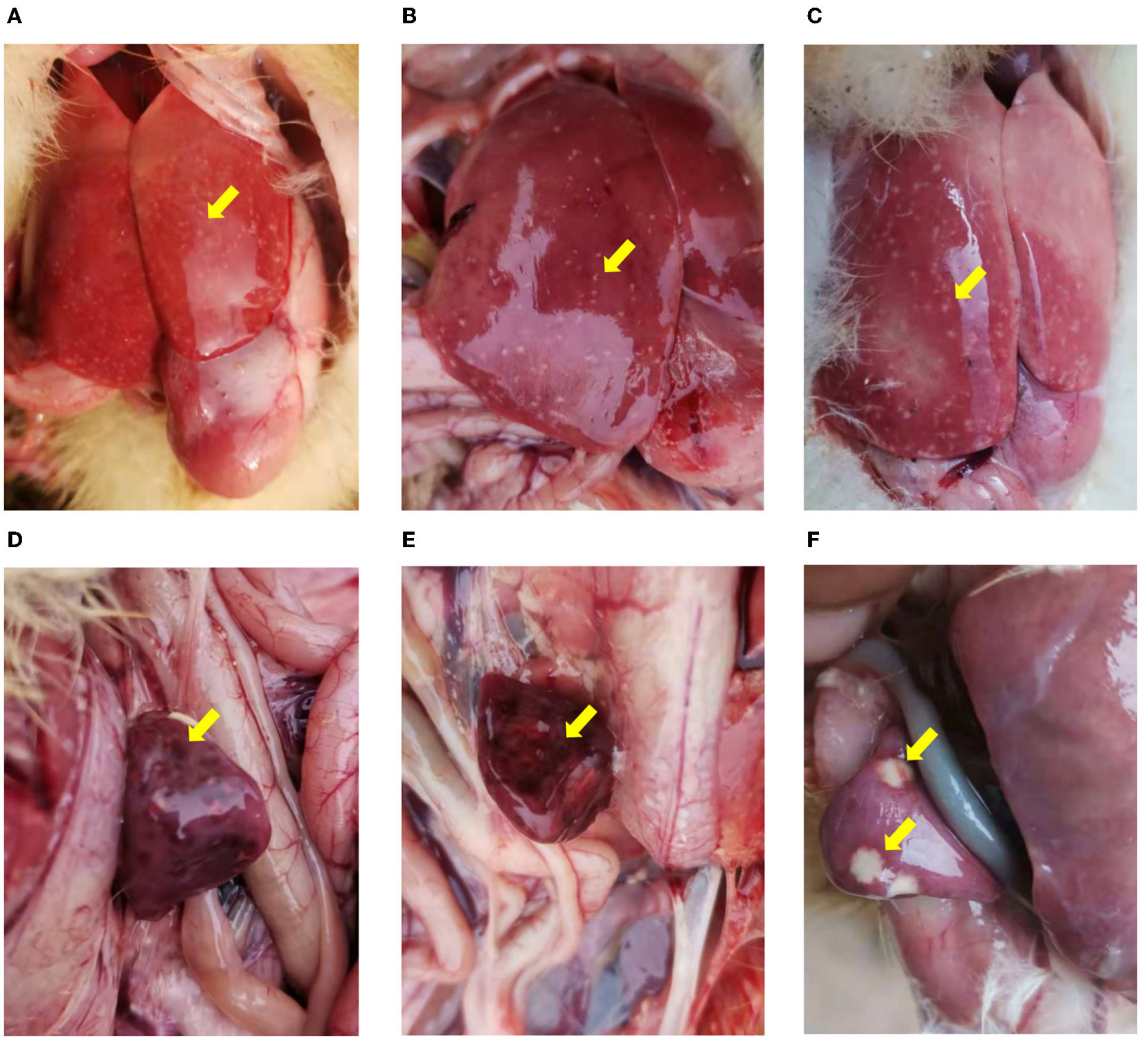
The pathological changes of the infected ducklings. **(A–C)** Hemorrhage and necrosis in the livers. **(D–F)** Various degrees of congestion and necrosis in the spleens. **(A, B, D, E)** Collected from Xinxiang city (case 1). **(C, F)** collected from Jiaozuo city (case 2).

For both NDRV outbreaks, most diseased ducklings were characterized by diarrhea, lethargy, increased eye discharge, loss of appetite, stunted growth, and paralysis. Among the organs collected from the dead ducklings in both cases, the predominant histologic lesions are located in the liver and spleen. The livers (Figures 1A–C) and spleens (Figures 1D–F) showed swelling, hemorrhage, and irregular necrosis in all dead ducklings. And other organs did not exhibit apparent lesions.

### 3.2. Molecular diagnosis

**Figure 2:**
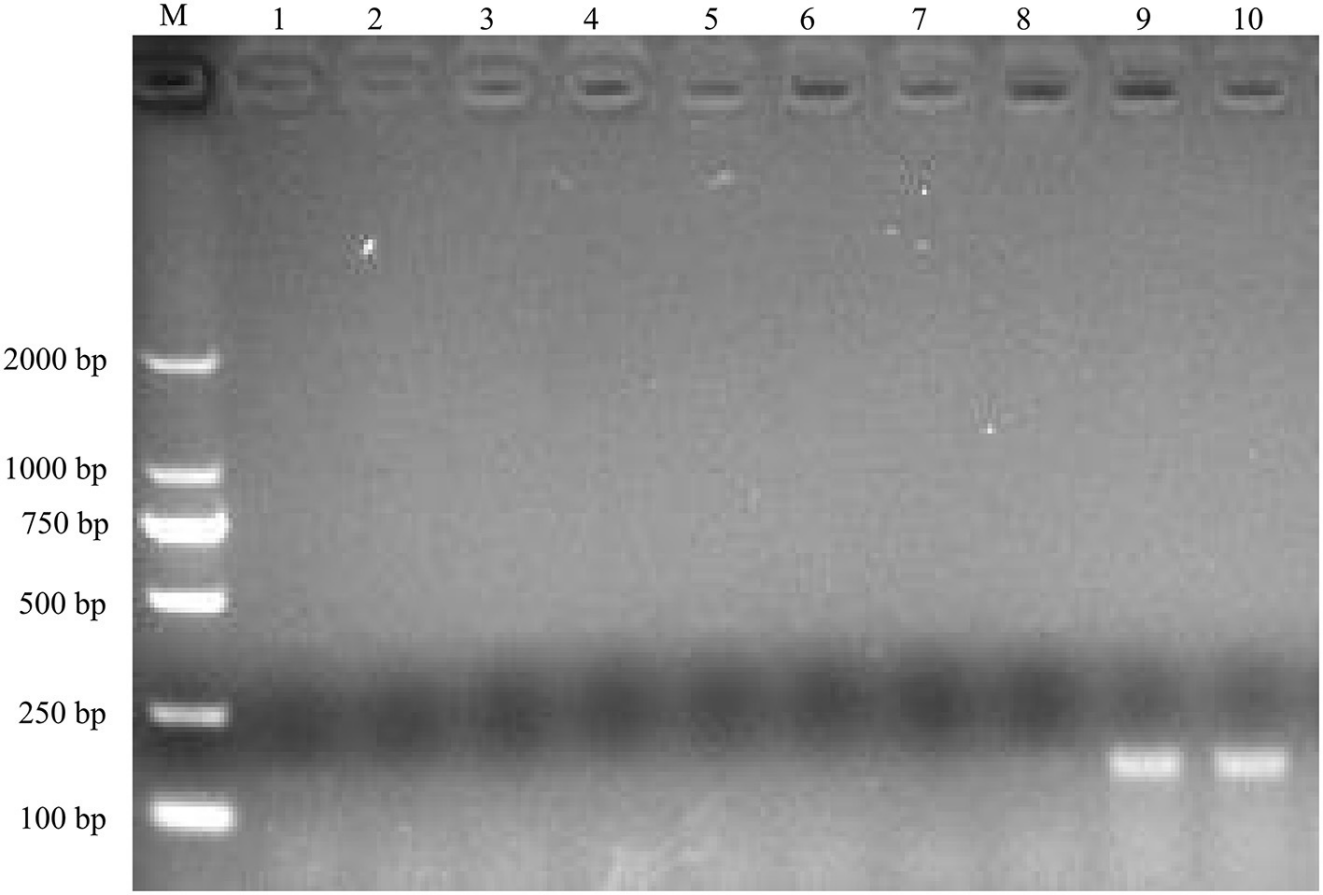
Agarose gel electrophoresis results for RT-PCR product of the potential causative agents. M, DL2000 DNA Ladder; lane 1, 2, MDRV; lane 3, 4, DHAV-1/DHAV-3; lane 5, 6, NDV, lane 7, 8, DTMUV; lane 9, 10, NDRV.

To identify the causative agent of the disease, RT-PCR assays were used to detect the potential viral pathogens. The samples from the dead duckling were positive for NDRV [The infection rate was 100% in case 1 (7/7) and case 2 (8/8)], and no corresponding nucleotide fragments were observed for MDRV, DHAV-1, DHAV-3, NDV, and DTMUV (Figure 2). In addition, *Riemerella anatipestifer* was also identified from four dead ducklings in case 2 by bacteria isolation.

### 3.3. Virus isolation

**Figure 3:**
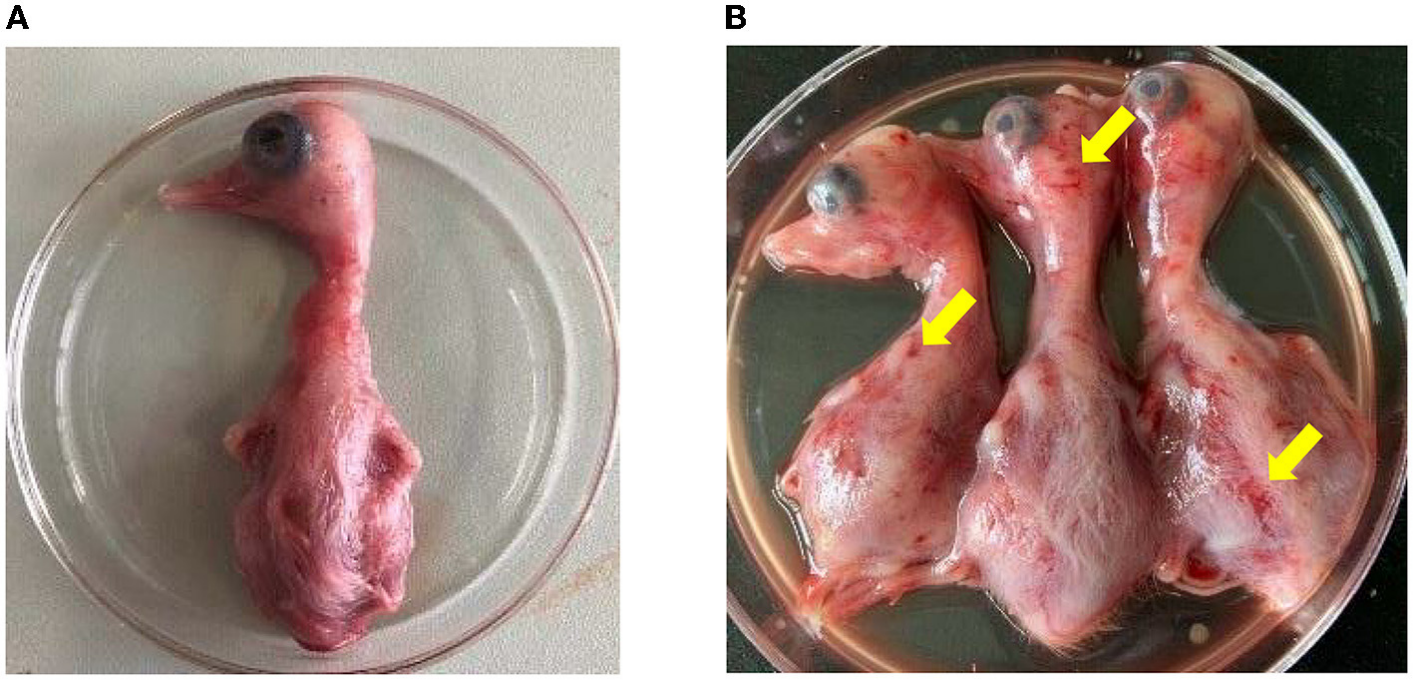
The pathogenicity of NDRV to duck embryos. **(A)** The duck embryos inoculated with PBS as the control. **(B)** The duck embryos inoculated with NDRV isolates in this study showed hemorrhage and oedema. The two duck embryos on the left and the third one in **(B)** were inoculated with NDRV isolated from Xinxiang city (case 1) and Jiaozuo city (case 2), respectively.

The homogenates of the positive liver and spleen samples were then inoculated into the allantoic cavity of 10-day-old healthy duck embryos. Compared with the duck embryos inoculated with PBS (Figure 3A), the inoculated duck embryos' bodies showed varying degrees of hemorrhage and dysplasia (Figure 3B). The allantoic fluids of the duck embryo inoculated with the samples were only positive for NDRV (Supplementary Figure S1). Eventually, two NDRV strains causing duck spleen necrosis disease were successfully isolated and termed as HNXX-1/2021 and HNJZ-2/2021, respectively.

### 3.4. Genetic and phylogenetic analysis

The σC genes of both newly identified NDRV isolates were 966 bp in length. To further investigate the genetic characteristics of both newly isolated NDRV strains, their σC genes and induced amino acid were compared with reference WRV strains. According to the sequence alignment of σC genes, Nucleotide identity between HNXX-1/2021 and HNJZ-2/2021 was 99.1%, which shared 98.4 and 98% identities with DRV/SY/Jiangsu/2018 in nucleotide, and 98.8 and 99.1% in deduced amino acid, respectively. Meanwhile, HNXX-1/2021 and HNJZ-2/2021 shared 98.4% and 98% in nucleotide, 98.5% and 98.8% in deduced amino acid with DRV/SDHZ17/Shandong/2017, respectively. The nucleotide sequences of the σC gene of HNXX-1/2021 and HNJZ-2/2021 were deposited into GenBank (Accession numbers: ON012751 and ON012752).

**Figure 4:**
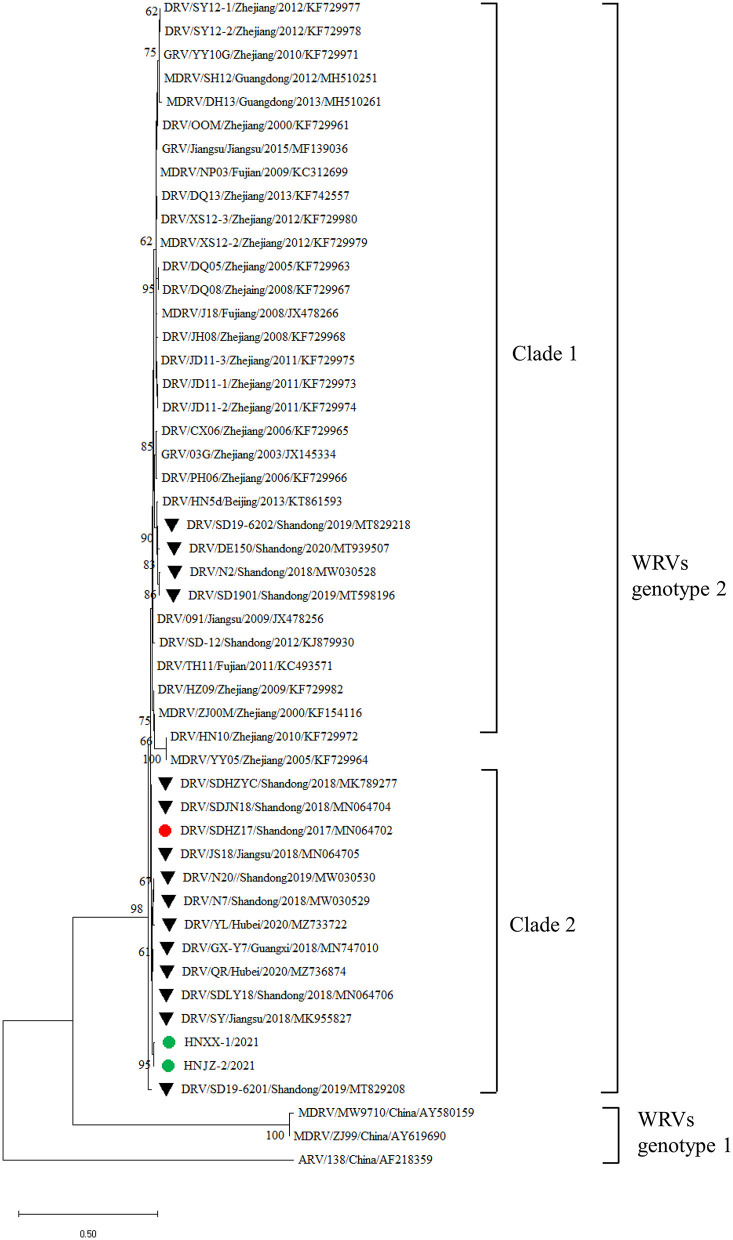
Phylogenetic tree was constructed based on σC protein amino acid sequences of DRVs. Numbers at nodes indicated bootstrap percentages obtained after 1,000 replicates. The σC protein amino acid sequence of a chicken-origin ARV/138/China strain (accession number AF218359) was included as an out-group. The green solid circles indicate the HNXX-1/2021 and HNJZ-2/2021 strains described in this study. The solid triangles indicate NDRV strains emerged after 2017. The province and time in the WRVs name represent the province and time at which the strain was collected, respectively.

To further explore the evolutionary characters of the newly identified NDRV strains, the phylogenetic tree of NDRVs was constructed based on the σC genes deduced amino acid sequences (Figure 4). As shown in the phylogenetic tree, HNXX-1/2021 and HNJZ-2/2021 were clustered together with DRV/QR/Hubei/2020, DRV/GX-Y7/Guangxi, DRV/SDLY18/Shandong/2018 and DRV/SY/Jiangsu/2018, which were NDRV strains emerged in recent years in China.

Remarkably, all the DRVs strains were clustered into two distinct clades. Clade 1 comprised the majority of DRVs strains that emerged before 2017, and few strains only emerged in Shandong province after 2017. Clade 2 comprised the majority of the NDRV strains that emerged after 2017, including the newly identified NDRV strains HNXX-1/2021 and HNJZ-2/2021 in this study.

### 3.5. Amino acid polymorphism analysis

To further explore the characteristics of both newly identified NDRV strains, amino acid polymorphism of the σC proteins was compared with reference strains (Supplementary Table S2). The results showed that most of the amino acid mutations in both newly identified NDRV isolates located in the σC protein head domain (Table 1), such as P233S, V253A, and A298V, indicating both isolates were closely related to each other. Meanwhile, some mutations were only found in HNXX-1/2021 and HNJZ-2/2021 (P233S in σC protein head domain), HeNXX-1/2021 (E305G, A328R, and T329Q in σC protein head domain) and HNJZ-2/2021 (A328R and T329Q in the σC protein head domain). Moreover, there was a unique amino acid mutation site (Q158H) in the σC protein shaft domain of both newly identified NDRV strains.

**Table 1:** Amino acid polymorphism of the σC protein among HNXX-1/2021, HNJZ-2/2021 and other WRVs strains.

**Amino**	**WRVs strains**						**Location**
**acid**	**Clade 2 within genotype2**				**Clade 2 within genotype2**		
**position**	**HNXX-1/2021**	**HNJZ-2/2021**	**Other**		**Isolated after 2017**	**Isolated before 2017**	
88	N	N	N/D		N	D	/
93	T^a^	T^a^	T^a^		S	S	/
132	A	A	S		A	S/A/T^e^	σC_shaft domain
138	R	R	R		R	R/Q	σC_shaft domain
158	H^b^	H^b^	Q		Q	Q/R^e^	σC_shaft domain
233	S^b^	S^b^	P		P	P	σC_head domain
253	A^a^	A^a^	V/A^a^		V	V	σC_head domain
298	V	V	V/A		V	A/T^c^	σC_head domain
305	G^c^	E	E		E	E	σC_head domain
328	R^c^	G^d^	A		A	A	σC_head domain
329	Q^c^	K^d^	T		T	T	σC_head domain

a Specific amino acid mutation only found in strains in clade 1.

b Specific amino acid mutation only found in HNXX-1/2021 and HNJZ-2/2021.

c Specific amino acid mutation only found in HNXX-1/2021.

d Specific amino acid mutation only found in HNJZ-2/2021.

e Tiny minority amino acids existed in the WRVs strains.

## 4. Discussion

ARV, MDRV, and NDRV are classified into the genus *Orthoreovirus*, members of the family *Reoviridae* (12, 18). ARV was first isolated from the respiratory tract of chickens suffering from chronic respiratory diseases in 1954 (28). MDRV was isolated from Muscovy ducklings for the first time in 1972 (7). In 2005, a novel duck reovirus disease was discovered in duck flocks in China, characterized by irregular hemorrhage and liver necrosis. The pathogen of this disease was isolated in 2011 and named NDRV distinguished from the classical MDRV (29). Since then, NDRV has become the predominant strain of reovirus that emerged in duck flocks in China (9, 13–15, 20, 23). Previous studies have demonstrated that NDRV has a broader host spectrum than MDRV (10, 15, 25), and different pathogenicity (14, 15, 24, 30, 31). In recent years, the diseases caused by NDRVs broke out in waterfowl flocks worldwide, have led to significant economic losses to the waterfowl industry. In this study, the both cases from Henan province showed swelling, hemorrhage, and irregular necrosis in livers and spleens in all diseased ducklings. The results of molecular diagnosis indicated that NDRV is the causative agent.

As the cell attachment protein of ARVs, σC proteins play an essential role in viral fusion, invasion, and pathogenicity (18–21). Additionally, the σC protein possesses the highest sequence variability among ARVs (18). The 18-aa deletion in the σC protein significantly enhanced the virulence of reovirus (30). Luo et al. indicated the NDRVs isolated in 2017 and thereafter clustered in a new subgroup (15). In this study, two NDRV isolates were identified, and the σC genes were amplified and sequenced. Further, Phylogenetic analysis based on the σC genes deduced amino acid sequences demonstrated that Chinese NDRVs had formed two distinct clades, with late 2017 as the turning point, suggesting that Chinese NDRVs have been evolving in different directions. More importantly, the vast majority of WRVs (29/33, 87.9%) within clade 1 were isolated before 2015, only 4 NDRV strains were isolated in Shandong province after 2017. Meanwhile, NDRV strains within clade 2 have emerged in the major duck production regions in China in recent years, such as Shandong, Henan, Jiangsu, Hubei, and Guangxi, and become predominant in duck flocks in China. Remarkably, four amino acid mutation sites located in the head domain, and one amino acid mutation site located in the shaft domain were discovered in both σC proteins of the newly identified NDRV strains. The role of these mutations changing the virulence is required further studies.

Previous studies have demonstrated that the genetic diversity of NDRV circulating in China is complex (3, 10, 13, 14, 25). Since using the attenuated MDRV vaccine in 2013, MDRV infection in waterfowl has significantly reduced in China in recent years. However, NDRV and N-MDRV emerged and spread widely in waterfowl in China (10, 15, 23, 25, 32), which caused significant economic losses in China. The complexity of epidemic strains undoubtedly facilitated virus mutation and recombination (14, 15), and complicated the genetic diversity of DNRV. To date, no effective NDRV vaccine is available, the complex genetic diversity of NDRV hampers the development of an effective vaccine. Though diverse vaccines against NDRV infection are under development, none is commercially available. The complex genetic diversity of NDRV poses more challenges to vaccine development, including poor cross-protection and the lack of markers for sero-surveillance.

With African swine fever outbreak in China in 2018, China's swine industry has suffered devastating destruction (33). After that, the waterfowl industry developed considerably in China. China has the largest waterfowl population worldwide. However, small-scale farms with poor biosecurity produce more than 70% of waterfowl in China, resulting in a higher risk of spreading NDRV. Furthermore, NDRV can spread vertically, as well as horizontally (34). Therefore, NDRV infection is much more challenging to prevent and control in duck production. Our findings will improve the molecular epidemiological picture of NDRV strain, facilitating understand its latest genetic evolution.

## 5. Conclusion

Two NDRV strains were isolated from duck farms during NDRV outbreaks in central China. Phylogenetic analysis revealed that Chinese NDRVs had formed two distinct clades. The both newly identified NDRV strains, which belong to clade 2, are current predominant strains. This study highlights the importance of continuous surveillance and evaluation of the epidemiology of NDRV in ducks. It improves the understanding of the genetic heterogeneity of NDRV in China, providing a foundation for developing effective prevention and control strategies for this persistent disease.

## Data availability statement

The datasets presented in this study can be found in online repositories. The names of the repository/repositories and accession number(s) can be found in the article/Supplementary material.

## Author contributions

ZP, XZ, and CB collected the samples and conceived and designed the experiments. ZP, HZ, and ZL performed the experiments. ZP, HQ, HW, and YL analyzed the data and wrote the paper. All authors have read and approved the final manuscript.
