# Supplementary material for: Identification and molecular characterization of novel duck reoviruses in Henan Province, China
Source: Front Vet Sci. 2023 Mar 30;10:1137967. doi: 10.3389/fvets.2023.1137967 (PMC10098080; doi:10.3389/fvets.2023.1137967)
Supplement: Supplementary file 1 [file Data_Sheet_1.docx]

Supplementary Material

Identification and Molecular Characterization of Novel Duck Reoviruses in Central China

Zhifeng Peng, Han Zhang, Xiaozhan Zhang, Haiyan Wang, Zihan Liu, Hongxing Qiao, Yujin Lv, Chuanzhou Bian**^*^**

*** Correspondence:** Chuanzhou Bian: chuanzhou-bian@126.com

# Supplementary Figures


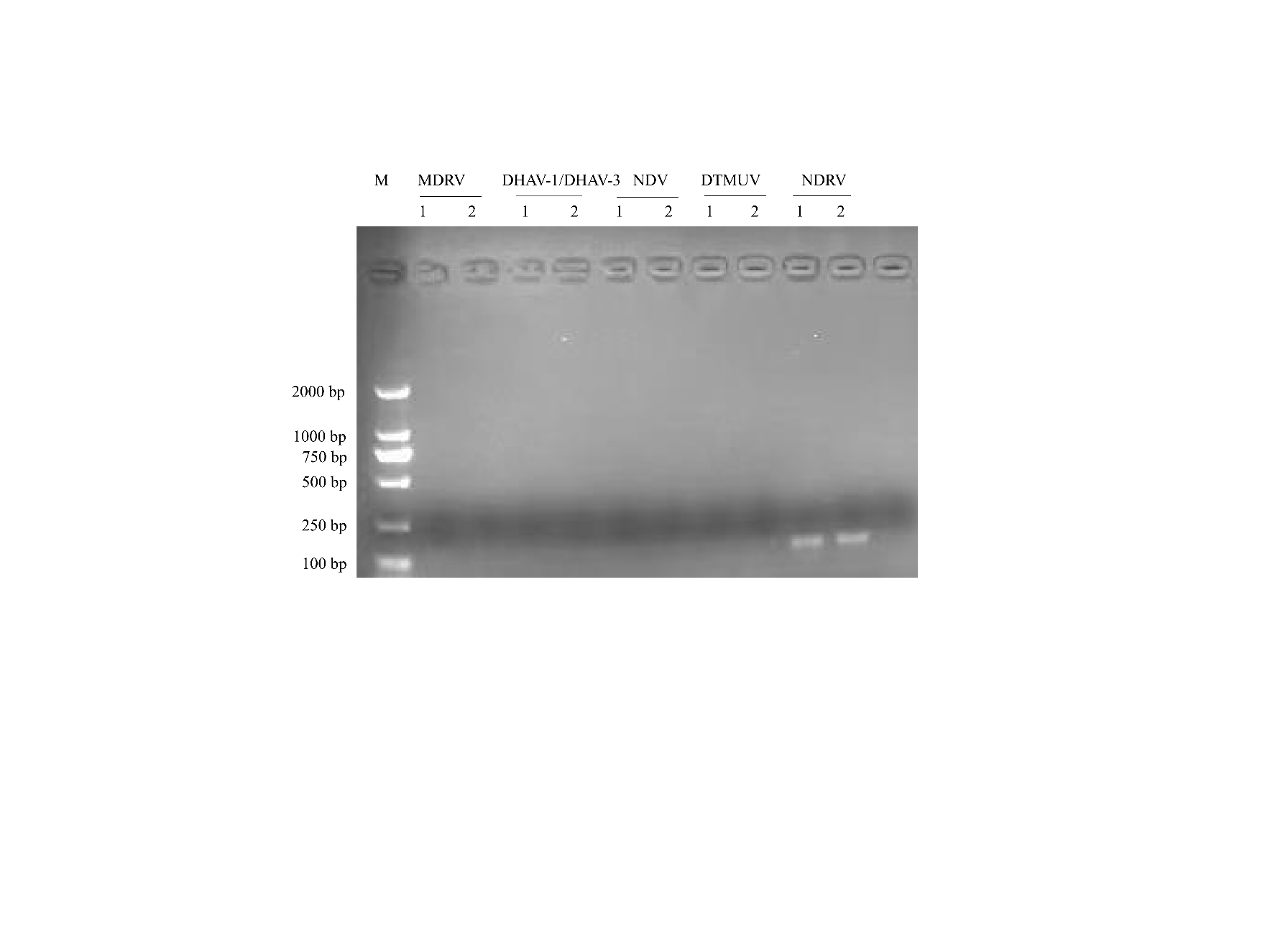


**Supplementary Figure S1. Molecular diagnosis of potential causative agents in the allantoic fluids by RT-PCR.**

Purified virus RNA was reversely transcribed into cDNA with random primers and then amplified for the potential causative agents in the allantoic fluids with specific primers, including MDRV (300 bp), DHAV-1 (492 bp), DHAV-3(286 bp), NDV (459 bp), DTMUV (382 bp) and NDRV (148 bp). 1 and 2 represented the different allantoic fluids inoculated with samples collected from case 1 and case 2, respectively.

# Supplementary Tables

Table S1 The primers used to detect potential viral pathogens

| Pathogens | Primers | Sequence (5'-3') | Annealing temperature (℃) | Product size (bp) |
| --- | --- | --- | --- | --- |
| MDRV | F | GAAAGCCGCTCAATCTCGGACCT | 53 | 189 |
|  | R | GCATCTATCTATCATACGCCATCCCGC |  |  |
| DHAV-1 | F | CAACTCGACCAATACCTGG | 60 | 492 |
|  | R | CCTGATGGACCATTGTAACTG |  |  |
| DHAV-3 | F | GAAATCTGCACTCAATGGAGAG | 60 | 286 |
|  | R | CCCAGGAAATGATTGGTCAG |  |  |
| NDV | F | AGCTGCAGGAATTGTAGTAA | 56 | 459 |
|  | R | TTAAACTGGTCATTGACAAA |  |  |
| DTMUV | F | GGAGAAGAAGGAAGAAGAAGT | 57 | 382 |
|  | R | ATGTCTGTTATTGGCGAGTT |  |  |
| NDRV | F | GTAAGCAACCTCAGGATATCG | 55 | 148 |
|  | R | TCATGTCGCGCGTCGTAT |  |  |

Table S2 The reference WRV strains in this study

| Strain | GenBank Accession | Region | Isolate Date | Strain | GenBank Accession | Region | Isolate Date |
| --- | --- | --- | --- | --- | --- | --- | --- |
| DE150 | MT93950 | Shandong | 2020 | NP03 | KC31269 | Fujiang | 2009 |
| DQ13 | KF742557 | Zhejiang | 2013 | HZ09 | KF729982 | Zhejiang | 2009 |
| SD19/6202 | MT829218 | Shandong | 2019 | CX09 | KF729969 | Zhejiang | 2009 |
| SD1901 | MT598196 | Shandong | 2019 | JH08 | KF729968 | Zhejiang | 2008 |
| SD19/6201 | MT829208 | Shandong | 2019 | PH06 | KF729966 | Zhejiang | 2006 |
| N20 | MW030530 | Shandong | 2019 | CX06 | KF729965 | Zhejiang | 2006 |
| N2 | MW030528 | Shandong | 2018 | DQ05 | KF729963 | Zhejiang | 2005 |
| GX-Y7 | MN747010 | Guangxi | 2018 | ZJ00M | KF154116 | Zhejiang | 2000 |
| SY | MK955827 | Jiangsu | 2018 | OOM | KF729961 | Zhejiang | 2000 |
| SDHZYC | MK789277 | Shandong | 2018 | JD11-3 | KF729975 | Zhejiang | 2011 |
| SDLY18 | MN064706 | Shandong | 2018 | TH11 | KC493571 | Fujian | 2011 |
| JS18 | MN06470 | Jiangsu | 2018 | JD11-2 | KF729974 | Zhejiang | 2011 |
| N7 | MW030529 | Shandong | 2018 | JD11-1 | KF72997 | Zhejiang | 2011 |
| SDHZ17 | MN064702 | Shandong | 2017 | HN10 | KF729972 | Zhejiang | 2010 |
| HN5d | KT861593 | Beijing | 2013 | 091 | JX478256 | Jiangsu | 2009 |
| SD-12 | KJ87993 | Shandong | 2012 | XS12-3 | KF729980 | Zhejiang | 2012 |
| XS12-2 | KF729979 | Zhejiang | 2012 | SY12-1 | KF729977 | Zhejiang | 2012 |
| 03G | JX145334 | Zhejiang | 2003 | DQ08 | KF729967 | Zhejiang | 2008 |
| J18 | JX478266 | Shandong | 2008 | Jiangsu | MF139036 | Jiangsu | 2015 |
| SH12 | MH510251 | Guangdong | 2012 | DH13 | MH510261 | Guangdong | 2013 |
| YY10G | KF729971 | Zhejiang | 2010 | YL | MZ733722 | Hubei | 2020 |
| QR | MZ736874 | Hubei | 2020 | YY05 | KF729964 | Zhejiang | 2005 |
